# Is Hydrogen Sulfide a Concern During Treatment of Lung Adenocarcinoma With Ammonium Tetrathiomolybdate?

**DOI:** 10.3389/fonc.2020.00234

**Published:** 2020-02-28

**Authors:** Xiang Li, Na Li, Li Huang, Shi Xu, Xue Zheng, Akil Hamsath, Mei Zhang, Lijun Dai, Hui Zhang, Justin Jong-Leong Wong, Ming Xian, Chun-tao Yang, Jinbao Liu

**Affiliations:** ^1^Affiliated Cancer Hospital & Institute of Guangzhou Medical University, Guangzhou, China; ^2^Guangzhou Municipal and Guangdong Provincial Key Laboratory of Protein Modification and Degradation, School of Basic Medical Science, Guangzhou Medical University, Guangzhou, China; ^3^Department of Pancreatobiliary Surgery, The First Affiliated Hospital of Sun Yat-sen University, Guangzhou, China; ^4^Department of Chemistry, Washington State University, Pullman, WA, United States; ^5^Epigenetics and RNA Biology Program Centenary Institute, The University of Sydney, Camperdown, NSW, Australia

**Keywords:** H_2_S, m^6^A methylation, Ammonium tetrathiomolybdate, lung cancer, PRPF6

## Abstract

Ammonium tetrathiomolybdate (ATTM) has been used in breast cancer therapy for copper chelation, as elevated copper promotes tumor growth. ATTM is also an identified H_2_S donor and endogenous H_2_S facilitates VitB_12_-induced S-adenosylmethionine (SAM) generation, which have been confirmed in m^6^A methylation and lung cancer development. The m^6^A modification was recently shown to participate in lung adenocarcinoma (LUAD) progression. These conflicting analyses of ATTM's anticancer vs. H_2_S's carcinogenesis suggest that H_2_S should not be ignored during LUAD's treatment with ATTM. This study was aimed to explore ATTM's effects on LUAD cells and mechanisms associated with H_2_S and m^6^A. It was found that treatment with ATTM inhibited cell growth at high concentrations, while enhanced cell growth at low concentrations in three LUAD cell lines (A549, HCC827, and PC9). However, another copper chelator triethylenetetramine, without H_2_S releasing activity, was not found to induce cell growth. Low ATTM concentrations also elevated m^6^A content in A549 cells. Analysis of differentially expressed genes in TCGA cohort indicated that m^6^A writer METTL3 and reader YTHDF1 were upregulated while eraser FTO was downregulated in LUAD tissues, consistent with the findings of protein expression in patient tissues. ATTM treatment of A549 cells significantly increased METTL3/14 and YTHDF1 while decreased FTO expression. Furthermore, inhibition of m^6^A with shMETTL3 RNA significantly attenuated eukaryotic translation initiation factor (eIF) expressions in A549 cells. Correlation analysis indicated that small nuclear ribonucleic protein PRPF6 was positively expressed with YTHDF1 in LUAD tissues. Knockdown of YTHDF1 partially blocked both basal and ATTM-induced PRPF6 expression, as well as A549 cell growth. Lastly, ATTM treatment not only raised intracellular H_2_S content but also upregulated H_2_S-producing enzymes. Exogenous H_2_S application mimicked ATTM's aforementioned effects, but the effects could be weakened by zinc-induced H_2_S scavenging. Collectively, H_2_S impedes ATTM-induced anticancer effects through YTHDF1-dependent PRPF6 m^6^A methylation in lung adenocarcinoma cells.

## Introduction

Ammonium tetrathiomolybdate (ATTM), with the formula (NH_4_)_2_MoS_4_, is a strong copper chelator. It has been clinically used in the treatment of copper toxicosis for Wilson's disease. At high concentrations, copper is also known to promote angiogenesis, metabolism and oxidative phosphorylation, thereby leading to tumor growth (1–3). Consequently, copper-chelating agents, like ATTM and triethylenetetramine (TETA), have been investigated in the treatment of cancers, including breast cancer, thyroid cancer and liver cancer (4–8). Additionally, copper-dependent enzyme, superoxide dismutase (SOD) 1, has been reported to facilitate lung adenocarcinoma (LUAD) development (9). Therefore, ATTM therapy may theoretically be extended to LUAD.

Interestingly, our lab and others have recently discovered ATTM is a pH-dependent hydrogen sulfide (H_2_S) donor (10, 11). There is no doubt that the development of ATTM as a therapeutic candidate must consider ATTM being both a copper chelator and a H_2_S releaser. It should be noted that the links between H_2_S and cancer development is still under debate (12). Some studies showed that elevated H_2_S can induce angiogenesis and tumor cell proliferation, thereby contributing to cancer development (13–15). H_2_S was also involved in VitB_12_-induced S-adenosylmethionine (SAM) generation (16), and high VitB_12_ has been found to raise the risk of lung cancer (17). Notably, in lung cancer tissues, endogenous H_2_S and its producing enzymes, like cystathionine beta-synthase (CBS), cystathionine gamma lyase (CSE, also known as CTH) and 3-mercaptopyruvate sulfurtransferase (3-MST), are highly expressed thereby benefiting cancer development (18, 19).

N^6^-Methyladenosine (m^6^A) methylation is one major type of mRNA modification, dynamically modulated by the corresponding writers, erasers and readers (20). The m^6^A methylation can differentially influence all fundamental aspects of mRNA metabolisms, including splicing, stability, and translation efficiency, when read by different m^6^A readers (21–23). Recently, the m^6^A methylation has been implicated in cancer pathogenesis, due to its induction of cell proliferation, invasion and immune disorders (24–28). Analysis of the cancer genome atlas (TCGA) cohort and recent studies (29, 30) indicated that m^6^A writer, methyltransferase like (METTL) 3 is upregulated in LUAD. The eraser, fat mass and obesity-associated gene (FTO), is downregulated. Both increased METTL3 and decreased FTO strongly suggest that LUAD tissues exhibit high m^6^A levels. However, no direct evidence has shown H_2_S can regulate m^6^A methylation, although H_2_S enhances SAM generation (16), a cofactor of METTL3/14 complex. We therefore hypothesize that m^6^A methylation is likely to be affected by H_2_S derived from ATTM and participate in LUAD development.

In the present study, we observed the effects of ATTM on lung adenocarcinoma cells and explored the roles of m^6^A in this process. Furthermore, we assessed the medication of H_2_S in ATTM-induced tumor cell proliferation, invasion and growth. Lastly, a purposive strategy was developed to overcome ATTM's side-effects in LUAD therapy.

## Materials and Methods

### Materials

ATTM and antibodies against METTL3 and METTL14 were purchased from Sigma-Aldrich Co. (St. Louis, MO, US). Antibodies against FTO and YTHDF1 were purchased from Abcam (Plc.Cambridge, MA, US). CuSO_4_ and Zn(OAc)_2_ were purchased from Meilun Biotechnology Co. (Dalian, China). TRIzol™ Regent (Invitrogen) was provide by Thermo Fisher Scientific Co. (Shanghai, China). Gemcell™ fetal bovine serum (FBS) was supplied by Gemini Company (Woodland, US).

### Cell Culture, Growth, Proliferation, and Invasion Assays

Lung adenocarcinoma cell lines (A549 and HCC827) were purchased from Cell Bank of Type Culture Collection of Chinese Academy of Sciences (Shanghai, China), and PC9 was obtained from ATCC. The cells were maintained in RPMI-1640 medium supplemented with 10% FBS at 37°C under an atmosphere of 5% CO_2_ and 95% air. They were passaged and harvested with 0.25% trypsin every other day.

Cell number was measured with Cell Counting Kit (CCK)-8 provided by Dojindo Lab (Kyushu, Japan). A549, HCC827, and PC9 cells were plated in 96-well plates at a density of 7, 000 cells/well. When grown to ~60–70% confluence, the cells were treated correspondingly. After the treatments, the CCK-8 solution (100 μL) at a 1:10 dilution with FBS-free medium was added to each well-followed by a 2-h incubation at 37°C. Absorbance (*A*) was measured at 450 nm with a microplate reader (Molecular Devices, US).

Cell proliferation was tested with BeyoClick™ 5-Ethynyl-2′-deoxyuridine (EdU) kit (Haimen, China). After the treatment of A549 cells with ATTM for 48 h, EdU incorporation assay was performed according to the manufacturer's instructions. TMB-derived color was measured at 630 nm with the microplate reader.

Cell invasion was observed with Transwell Migration Assay, as described (31) with modifications. RPMI-1640 medium (1% FBS) containing ATTM or Na_2_S in the absence or presence of Zn(OAc)_2_ was added to the lower chambers of the 12-well format transwells (8 μm-pore, BD Biosciences). A549 cells were seeded in the upper chambers at 10^5^ cells per well, following a 48 h-culture. After that, the transwells were fixed in methanol, and stained with Giemsa solution. The unmigrated cells were removed from the top of the membranes using cotton swabs. To quantify the number of migrated cells in the bottom of the membrane, four random images of each group were taken at 10× under a light microscope. Migrated cell number was counted with Image J software.

### Quantification of m^6^A RNA Methylation

After treatments of A549 cells with increasing concentrations of ATTM for 24 h, total RNA was extracted using TaKaRa MiniBest kits (Kusatsu, Japan) and quantitated with NanoDrop 1000 spectrophotometer (Thermo Fisher, US). The m^6^A RNA was detected with EpiQuik m^6^A RNA Methylation Colorimetric kit (Farmingdale, US). Briefly, 200 ng of fresh extracted RNA sample was added into strip wells with RNA high binding solution, and incubated for 90 min at 37°C. After three washes, capture antibody, detection antibody, and enhancer solution were applied in turn, and incubated for 1 h at 37°C. After washes, color developing solution was added and incubated for 6 min in the dark. When the solution became blue in the m^6^A positive wells, stop solution was added to turn the color into yellow. Lastly, the absorbance of stable yellow was measured at 450 nm with the microplate reader.

### Western Blotting for Protein Expression

After exposed to 60 μM ATTM for 24 h, A549 cells were collected and split at 4°C. Total proteins in the lysate were quantitated with a BCA kit. Thirty micrograms of total protein sample were loaded in SDS-PAGE and electrophoresed. At the end of electrophoresis, the proteins were transferred to PVDF membranes. The membranes were blocked with 5% fat-free milk in Tris-base buffered saline containing 0.1% Tween-20 (TBS-T) for 1 h at room temperature, and then incubated with the primary antibodies against m^6^A related proteins (METTL3, METTL14, FTO, and YTHDDF1), and H_2_S-producing enzymes (CSE, CBS, and MPST), respectively, with gentle agitation overnight at 4°C. After three washes, corresponding HRP-conjugated secondary antibodies were applied and incubated for 1.5 h at room temperature. The signal was visualized using an enhanced chemiluminescence detection system. The intensity of bands was quantified with Image J software.

### Analysis of TCGA Database and Measurement of Protein Expression of LUAD Patients

Transcriptional expressions of m^6^A or H_2_S related genes in primary tumor tissues and adjacent tissues of LUAD patients, as well as correlation analysis in tumor tissues, were performed through TCGA cohort research tool (*UALCAN*). Furthermore, the protein expressions of METTLE3, METTL14, and FTO in LUAD patients were verified with Western blotting assay as above.

### Gene Knockdown

Gene expression microarray data were downloaded at https://www.ncbi.nlm.nih.gov/geo/query/acc.cgi?acc=GSE76367 (29). METTL3 was knocked down through short hairpin (sh) RNA in A549 cells, and gene expression was profiled by high throughput sequencing. Selected genes of eukaryotic translation initiation factors (eIFs) were shown as the maximum *vs*. minimum of the ratio shMETTL3 to shGFP.

Small interfering RNA (siRNA) against human YTHDF1 (Gene ID: 54915) was synthesized by GenePharma Co., Ltd (Shanghai, China). YTHDF1 siRNA and control random non-targeting siRNA were transfected into the A549 cells using Lipofectamine 2000 (Invitrogen, USA) (32). To raise the transfection efficiency, the cells were incubated with 20 nM YTHDF1 siRNA or Control siRNA for 6 h followed by a 24-h culture. The silencing ability was evaluated by Western blotting assay.

### Quantitative Polymerase Chain Reaction for PRPF6 Gene Expression

Total RNAs were extracted from A549 cells and quantitated as above. First-strand cDNA was synthesized using TaqMan SYBR® Premix Ex TaqTM II (Tli RNase H Plus) virus reverse transcriptase and 2 μg RNA template in 20 μL reaction volume. The cDNA was used for real-time PCR with Prime Script RT reagent kit with gDNA Eraser (Life Technologies, US). Actin was used to normalize the expression of PRPF6. The results were analyzed using the comparative Ct method (2-ΔΔCt with logarithmic transformation). Primer sequences were displayed as below: PRPF6 forward 5-GTCATGCGTGCCGT GATTG-3 and reverse 5-TCCAGGGCATTGTGGGCTA-3, Actin forward 5-TGGCACCCAGCACAATGAA-3 and reverse 5-CTAAGTCATAGTCCGCCTAGAAGCA-3. PCRs were carried out as follows: initial denaturation at 95°C for 30 s, 40 cycles of 95°C for 5 s, 60°C for 34 s, and 95°C for 15 s, 60°C for 1 h, followed by a final extension at 95°C for 15 s.

### Measurement of H_2_S Levels

ATTM-mediated H_2_S generation in cells was determined with a H_2_S fluorescent probe WSP-5 (33). A549 cells were inoculated in 24-well plates and grown to 60~70% confluence. After treated with ATTM, the cells were incubated with 10 μM WSP-5 in 1% FBS medium at 37°C for 30 min in the dark. Cell imaging was carried out after a slight wash with PBS. The intracellular H_2_S-triggered fluorescence was visualized under AMG fluorescence microscopy (Advanced Microscopy Group, US).

For H_2_S scavenging induced by Zn(OAc)_2_, CuSO_4_, FeCl_3_ or VitB_12_ was observed through a Unisense H_2_S micro-sensor (Tueager 1, Denmark) (34). Into 5 mL PBS buffer, fresh Na_2_S stock solution was added to produce a 100 μM Na_2_S solution. When the curve reached the peak and kept stable, the same dose of above compounds was immediately added, respectively. The curves were recorded correspondingly with the H_2_S sensor. For Zn(OAc)_2_ or CuSO_4_-induced continuous H_2_S scavenging from ATTM solution was recorded for 6 h. Into 20 mL of 500 μM ATTM solution (pH 5), fresh Zn(OAc)_2_ or CuSO_4_ stock solutions was added, respectively, to produce a 250 μM solution. Real-time H_2_S content was monitored with the H_2_S sensor and quantified against a standard curve.

### Statistical Analysis

The experiment data are presented as means ± standard deviation (SD). Significance between groups was evaluated by one-way analysis of variance (ANOVA) followed by *Student-Newman-Keuls* test using GraphPad Prism 8 software (San Diego, US). A probability <0.05 was considered statistically significant.

## Results

### Low ATTM Levels Enhanced Growth of Lung Adenocarcinoma Cells

**Figure 1:**
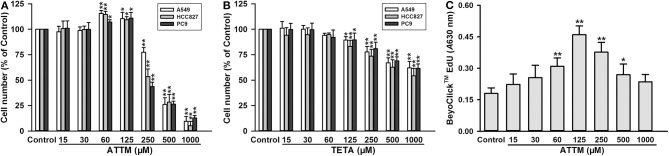
Effects of copper chelators on LUAD cell growth and proliferation. After treatment with increasing concentrations of ATTM **(A)** or TETA **(B)** for 48 h, cell number of three cell lines (A549, HCC827, and PC9) was counted with CCK-8 assay. **(C)** After ATTM treatment for 48 h, the proliferation of A549 cells was tested with BeyoClick™ EdU kit. Data are expressed as mean ± SD of four independent experiments. *P < 0.05, **P < 0.01 vs. Control group.

As shown in Figure 1A, treatment with high concentrations (≥250 μM) of ATTM for 48 h remarkably reduced cell number, in three types of lung adenocarcinoma cells (A549, HCC827, and PC9). However, at low concentrations (from 60 to 125 μM), the treatment distinctively increased cell number. Notably, another copper chelator TETA, without H_2_S releasing activity, was not found to elevate cell number at the same treatment profile (Figure 1B). Furthermore, 60–125 μM of ATTM treatment could also induce A549 cell proliferation as evaluated by EdU assay (Figure 1C). The result indicates that low ATTM concentrations are able to promote lung adenocarcinoma growth.

### ATTM Induced mRNA m^6^A Methylation in Lung Adenocarcinoma A549 Cells

**Figure 2:**
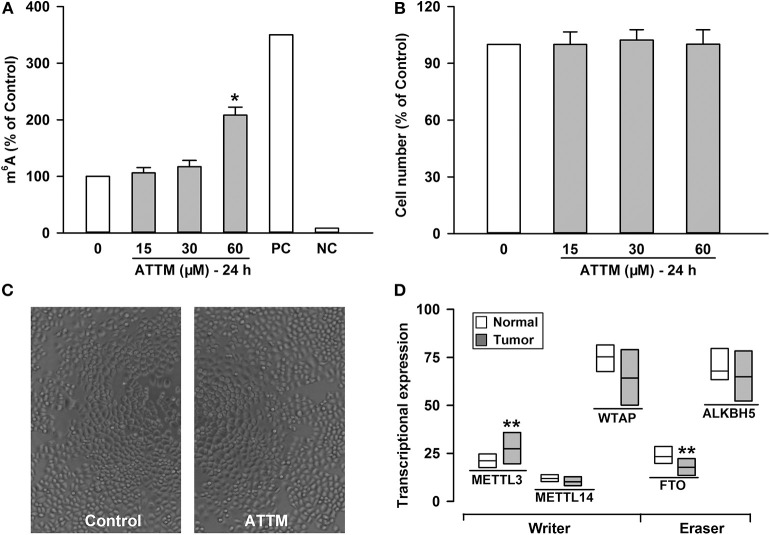
Effects of ATTM on mRNA m^6^A methylation in A549 cells. The cells were treated with ATTM at concentrations ranging from 0 to 60 μM for 24 h. **(A)** The content of m^6^A mRNA was measured with a commercial kit (PC, positive control; NC, negative control). **(B)** The cell number was tested with CCK-8 assay. Data are presented as mean ± SD. *n* = 4. *P < 0.01 vs. Control group. **(C)** Growth of quiescent A549 cells and cells exposed to 60 μM ATTM for 24 h were captured using digital microphotograph. **(D)** Expressions of the m^6^A writers (METTL3, METTL14, and WTAP) and erasers (FTO and ALKBH5) were analyzed between LUAD primary tumor tissues (*n* = 515) and normal tissues (*n* = 59) in TCGA cohort. Data are showed as median ± quartile. **P < 10^−4^ vs. Normal tissues.

To understand why ATTM enhanced lung adenocarcinoma growth, A549 cells were selected as a representative in the following experiments. Since mRNA m^6^A methylation is involved in a variety of cancer growth including lung adenocarcinoma, intracellular m^6^A mRNA level was then investigated. As shown in Figure 2A, the m^6^A mRNA content was significantly elevated after the exposure of A549 cells to 60 μM ATTM for 24 h. However, the treatment duration (half of 48 h) did not alter cell number (Figure 2B) or growth status (Figure 2C). Notably, analysis of TCGA cohort shows that LUAD condition significantly upregulates the m^6^A writer METTL3, while downregulates the m^6^A eraser FTO (Figure 2D). The result indicates that ATTM can trigger mRNA m^6^A methylation before cell growth in lung adenocarcinoma cells.

### Upregulated m^6^A Methylation Was Involved in LUAD Progression and ATTM-Induced mRNA Translation in Lung Adenocarcinoma A549 Cells

**Figure 3:**
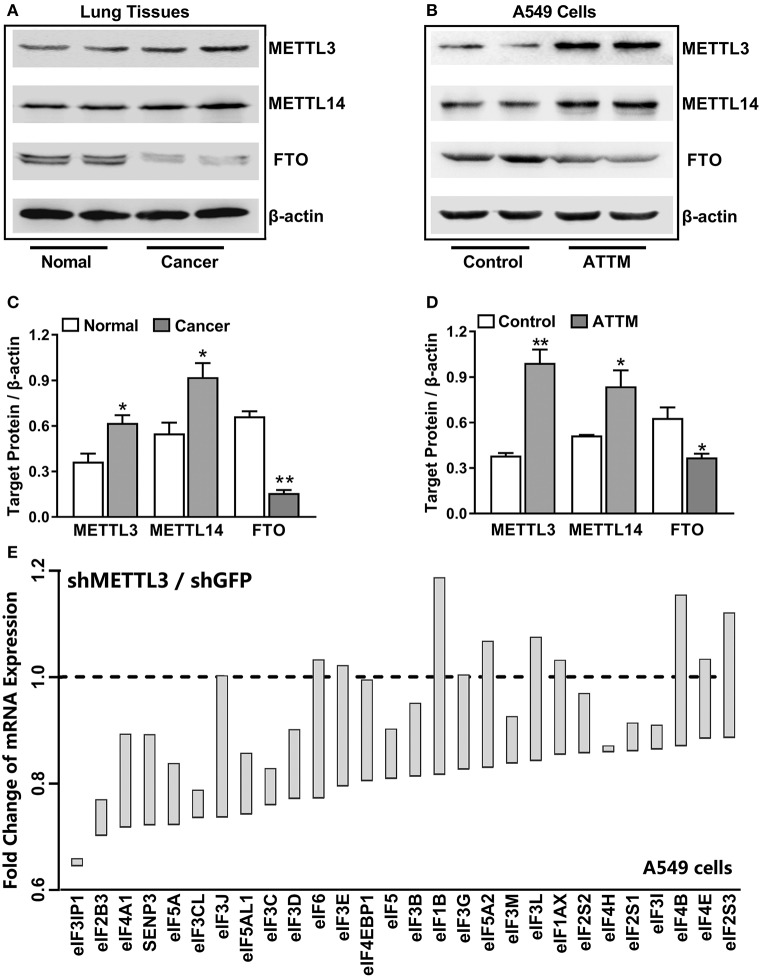
Effects of lung cancer condition and ATTM treatment on m^6^A related protein expression. **(A)** Confirmed LUAD patient tumor tissues and normal adjacent tissues, as well as **(B)** A549 cells treated with 60 μM ATTM for 24 h and normal cells, were collected and split. Total proteins were used for Western blot assay to measure the expressions of METTL3, METTL14, and FTO. Corresponding quantifications were shown as **(C,D)**, respectively. Data are presented as mean ± SD. *n* = 2. *P < 0.05, **P < 0.01 vs. Control group. **(E)** A549 cells transfected with shMETTL3 or shGFP were collected and gene expression was profiled by high throughput sequencing. Representative genes of eukaryotic translation initiation factors (eIFs) were analyzed and shown as the maximum *vs*. minimum of the ratio of shMETTL3 to shGFP (GSE76367).

To uncover the mechanisms underlying the increased m^6^A mRNA levels in ATTM-treated cells, m^6^A related proteins were detected with Western blot. As shown in Figures 3A,C, comparing with the adjacent tissues, the writer (METTL3 and METLL14) expressions were upregulated in tumor tissues of LUAD patients, while the eraser FTO expression was downregulated. Importantly, the writer (METTL3 and METLL14) expressions in A549 cells could also be upregulated, while the eraser FTO could be downregulated under ATTM treatment (Figures 3B,D), supporting the finding of the increased intracellular m^6^A content. To confirm the roles of m^6^A methylation, we investigated gene expression profile in METTL3 knockdown A549 cells. As shown in Figure 3E, shRNA-mediated METTL3 knockdown significantly inhibited the expressions of translation initiation factors, including eIFs (2B3, 3B, 3C/CL, 3D, 3IP1, 4A1, and 5/5A). This suggests that m^6^A methylation is necessary to translation process in A549 cells, and that the elevated m^6^A levels may be important for LUAD progression and ATTM-induced tumor growth.

### YTHDF1 Mediated ATTM-Induced Growth in Lung Adenocarcinoma A549 Cells

**Figure 4:**
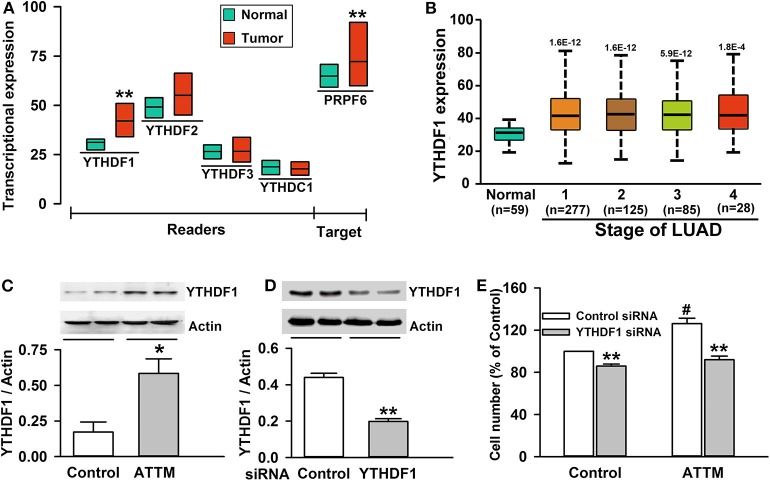
Roles of YTHDF1 in ATTM-induced growth of A549 cells. **(A)** Expressions of m^6^A readers (YTHDF1, YTHDF2, YTHDF3, and YTHDC1) and a potential target gene (PRPF6) were compared between LUAD patient primary tumor tissues (*n* = 515) and normal tissues (*n* = 59) in TCGA cohort. Data are shown as median ± quartile. **P < 10^−7^ vs. Normal tissues. **(B)** YTHDF1 gene expression in normal tissues and various LUAD stage tissues was analyzed in TCGA cohort. Data are shown in the box plot. **(C)** After treatment of A549 cells with 60 μM ATTM for 24 h, YTHDF1 protein expression was measured with Western blot assay, and the densitometric analysis was performed. Data are shown as mean ± SD. *n* = 2. *P < 0.05 vs. Control group. **(D)** A549 cells were incubated with YTHDF1 siRNA or Control siRNA for 6 h followed by a 24-h culture, and the interference efficiency was determined with Western blot assay. **(E)** Normal A549 cells and YTHDF1-knocked down cells were treated with 60 μM ATTM for 48 h, and cell counting assay was performed. Data are presented as mean ± SD. *n* = 4. **P < 0.01 vs. Control siRNA cells, ^#^*P* < 0.01 vs. ATTM-free cells.

Although the increased m^6^A writer METTL3/14 and the decreased eraser FTO can result in the enhancement of m^6^A content, it is the m^6^A readers that directly determine the outcome of m^6^A methylated mRNA. With TCGA cohort, we examined the four common m^6^A readers (YTHDF1, YTHDF2, YTHDF3, and YTHDC1) in LUAD tumor samples and normal samples, and found YTHDF1 highly expressed in tumor tissues (Figure 4A). The increased YTHDF1 continuously expressed within various stages of LUAD (Figure 4B). Importantly, it was found that treatment of A549 cells with 60 μM ATTM for 24 h remarkably upregulated YTHDF1 protein expression (Figure 4C). After knockdown of YTHDF1 with siRNA in A549 cells (Figure 4D), both basal and ATTM-triggered cell growth were attenuated (Figure 4E).

### YTHDF1-Mediated Cell Growth Was Associated With PRPF6 Induction in ATTM-Treated A549 Cells

**Figure 5:**
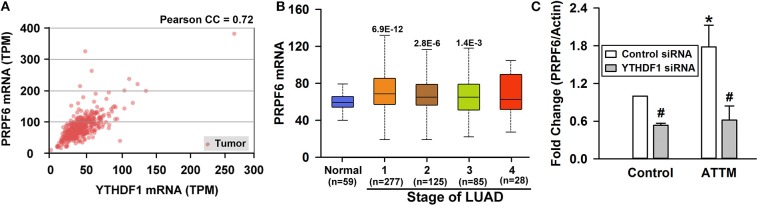
Identification of PRPF6 as a target gene of YTHDF1. **(A)** TCGA-based correlation analysis between PRPF6 and YTHDF1. **(B)** PRPF6 gene expression in normal tissues and various LUAD stage tissues was analyzed through TCGA cohort. Data are shown in the box plot. **(C)** Normal A549 cells and YTHDF1-knocked down cells were treated with 60 μM ATTM and then mRNA was extracted for analysis of PRPF6 expression with qPCR. Data are shown as mean ± SD. *n* = 4. *P < 0.01 vs. ATTM-free cells. ^#^*P* < 0.01 vs. Control siRNA cells.

To explore targets involved in YTHDF1-mediated A549 cell growth, we screened genes that are positively correlated with YTHDF1 in TCGA LUAD cohort. As shown in Figure 5A, PRPF6 was found to be positively expressed with YTHDF1 in LUAD tissues (*r* = 0.72). Similar to YTHDF1's expression profile, the increased PRPF6 expression lasted various stages of LUAD (Figure 5B). Furthermore, qPCR analysis showed that PRPF6 mRNA level was significantly reduced after YTHDF1 knockdown in A549 cells. Additionally, the knockdown attenuated ATTM-induced PRPF6 mRNA expression (Figure 5C). The result reveals that PRPF6 may be a potential target gene involved in YTHDF1-mediated A549 cell growth.

### H_2_S Was Significant to ATTM-Induced A549 Cell Growth and m^6^A Methylation

**Figure 6:**
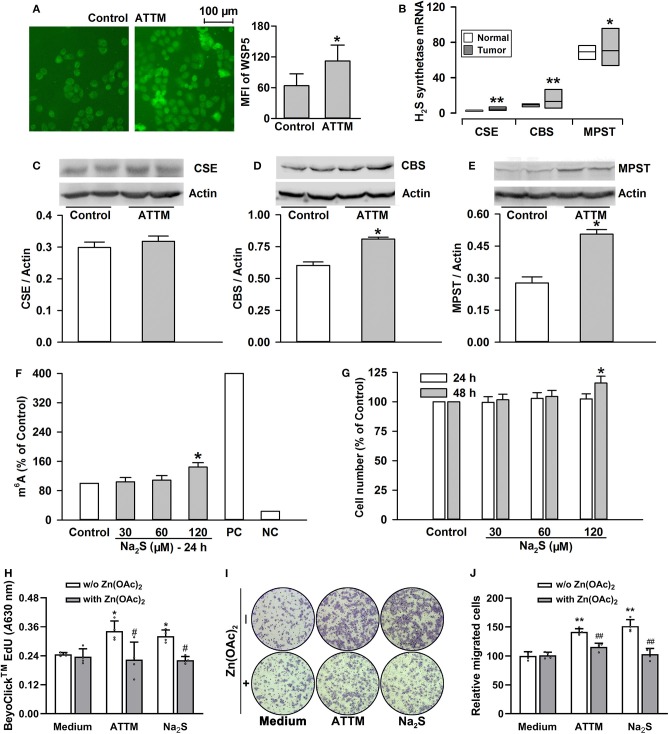
Roles of H_2_S in ATTM-induced m^6^A methylation and growth in A549 cells. **(A)** A549 cells were treated with normal medium (Control) and 60 μM ATTM for 3 h. Intracellular H_2_S content was observed with H_2_S fluorescent probe WSP5 staining followed by fluorescence photography. **(B)** Expressions of H_2_S synthetases (CSE, CBS and MPST) were compared between LUAD patient primary tumor tissues (*n* = 515) and normal tissues (*n* = 59) in TCGA cohort. Data are showed as median ± quartile. *P < 10^−6^, **P < 10^−11^ *vs*. Normal tissues. **(C–E)** After treatment of A549 cells with 60 μM ATTM for 24 h, the expressions of CSE **(C)**, CBS **(D)**, and MPST **(E)** were measured with Western blot, and then densitometric analysis were performed. Data are shown as mean ± SD. *n* = 2. *P < 0.05 *vs*. Control. **(F)** A549 cells were treated with increasing concentrations of Na_2_S for 24 h, intracellular m^6^A mRNA was tested with a commercial kit. **(G)** A549 cells were treated with the indicated concentrations of Na_2_S for 24 and 48 h, respectively. The cell number was measured with CCK-8 assay. **(H–J)** A549 cells were treated with 60 μM ATTM or 120 μM Na_2_S in the absence or presence of 25 μM Zn(OAc)_2_ for 48 h. Cell proliferation was tested with EdU incorporation assay **(H)**. Cell invasion was observed with Transwell Migration Assay **(I)** and migrated cells were counted using ImageJ software. Data are presented as the mean ± SD. *n* = 4. *P < 0.05, **P < 0.01 *vs*. Control/Medium. ^#^*P* < 0.05, ^##^*P* < 0.01 *vs*. Zn(OAc)_2_ free group.

ATTM was previously demonstrated to generate H_2_S on acid conditions in our lab. As shown in Figure 6A, the exposure of A549 cells to ATTM markedly raised intracellular H_2_S levels. With TCGA cohort, we studied endogenous H_2_S synthetase expressions and, found that CSE/CTH, CBS and MPST expressions were enhanced in tumor tissues comparing with normal samples (Figure 6B). Additionally, the exposure of A549 cells to 60 μM ATTM for 24 h obviously upregulated CBS and MPST expressions (Figures 6D,E), however, remarkable change of CSE was not found (Figure 6C). Notably, direct H_2_S donation (Na_2_S) could also induce m^6^A methylation (Figure 6F), as well as cell growth (Figure 6G), proliferation (Figure 6H) and invasion (Figures 6I,J), indicating H_2_S being an effector in ATTM-induced m^6^A methylation and cell growth.

### H_2_S Scavenging Attenuated ATTM-Induced A549 Cell Growth and m^6^A Methylation

**Figure 7:**
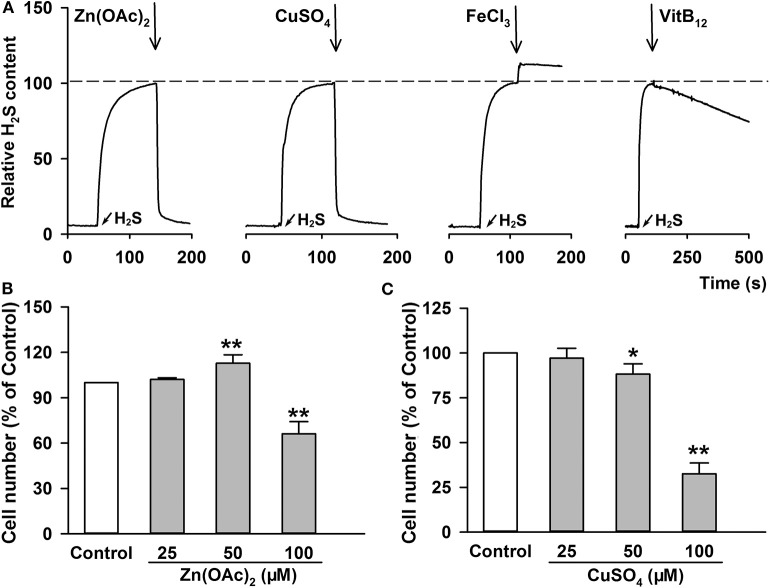
H_2_S removing efficiency and cell toxicity of the indicated H_2_S scavengers. **(A)** After adding 100 μM Na_2_S, the same amount of indicated H_2_S scavengers, including Zn(OAc)_2_, CuSO_4_, FeCl_3_ and VitB_12_, were added, respectively. The content of H_2_S molecule was monitored with a Unisense H_2_S microsensor. **(B–C)** A549 cells were treated with increasing concentration of Zn(OAc)_2_ **(B)** or CuSO_4_ **(C)** for 24 h. The cell number was measured with CCK-8 assay. Data are shown as the mean ± SD. *n* = 4. *P < 0.05, **P < 0.01 *vs*. Control group.

Since the enhanced H_2_S generation was involved in ATTM-induced A549 cell growth and m^6^A methylation, scavenging H_2_S might overcome these side-effects of ATTM. Through testing several common H_2_S scavengers, it was found that Zn(OAc)_2_ and CuSO_4_ had powerful ability to remove H_2_S, while the ability of VitB_12_ and FeCl_3_ was weak or even undetectable (Figure 7A). Additional cell viability examination showed that both Zn(OAc)_2_ and CuSO_4_ were non-toxic at concentrations <25 μM (Figures 7B,C).

**Figure 8:**
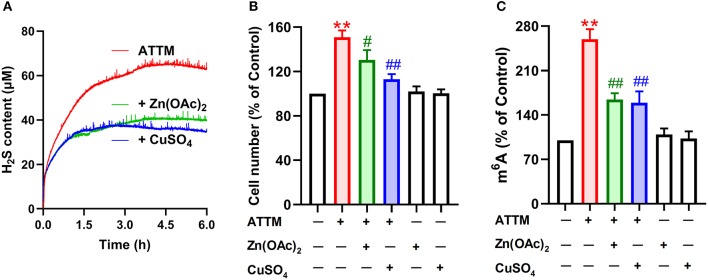
Effects of H_2_S scavengers on ATTM-induced A549 cell growth and m^6^A methylation. **(A)** ATTM solutions were prepared in the presence or absence of 25 μM Zn(OAc)_2_ or CuSO_4_. Subsequently, released H_2_S was recorded for 6 h with a H_2_S micro-sensor. **(B)** A549 cells were treated with 60 μM ATTM for 48 h in the absence or presence of 25 μM Zn(OAc)_2_ or CuSO_4_. The cell number was measured with CCK-8 assay. **(C)** A549 cells were treated with 60 μM ATTM for 24 h in the absence or presence of 25 μM Zn(OAc)_2_ or CuSO_4_. Intracellular m^6^A mRNA was tested with a commercial kit. Data are shown as the mean ± SD. *n* = 4. **P < 0.01 *vs*. Control. ^#^*P* < 0.05, ^##^*P* < 0.01 *vs*. ATTM alone group.

To make sure H_2_S is necessary to ATTM-induced biological process in A549 cells, we observed the scavenging effect of Zn(OAc)_2_ or CuSO_4_ on ATTM-induced H_2_S release within a period of 6 h. As shown in Figure 8A, adding 25 μM Zn(OAc)_2_ or CuSO_4_ time-dependently attenuated ATTM-induced H_2_S release. Notably, application of 25 μM Zn(OAc)_2_ inhibited ATTM-induced cell growth (Figure 8B), proliferation (Figure 6H) and invasion (Figures 6I,J). More importantly, Zn(OAc)_2_ was also able to attenuate ATTM-induced m^6^A methylation (Figure 8C). Collectively, the result suggests that H_2_S is necessary and sufficient to ATTM-induced biological changes, and scavenging H_2_S can potentially overcome ATTM's side-effects in lung cancer treatment.

## Discussion

In the present study, ATTM was found to promote cell growth, proliferation and invasion in lung adenocarcinoma cells, through YTHDF1-dependent PRPF6 m^6^A methylation. H_2_S was involved in the above effects of ATTM. Importantly, scavenging H_2_S was proved to overcome the side-effects of ATTM in lung cancer therapy.

Copper ions are known to essentially maintain organism functions by regulating activity of key enzymes, like cytochrome C oxidase and SOD. However, its aberrant increase in plasma usually leads to pathological consequences, including Wilson's disease and cancers. During cancer development, high contents of copper are believed to enhance angiogenesis and blood supply, as well as activity of mitochondrial cytochrome C oxidase, thereby promoting solid tumor growth and metastasis (1–3, 35). Consequently, chelating agents of copper ions, like ATTM and TETA, have become promising anticancer drugs. The clinical trials of ATTM in breast cancer have recently made great progress (4–6). Notably, SOD1, a copper-dependent enzyme, was reported to participate in lung cancer growth and has become a significant therapeutic target (9). Therefore, ATTM may exert anticancer effects in lung adenocarcinoma through reduction of copper ions. In this study, at high concentrations, ATTM could indeed inhibit the growth of lung adenocarcinoma cells. However, at low concentrations, ATTM distinctively promoted tumor cell growth, as well as proliferation and invasion. The result was different from the reported effects of ATTM in breast cancer (6) and BRAF-driven papillary thyroid cancer (8), which suggests that the findings in other cancers should not be simply transplanted to lung cancer.

To uncover the mechanisms underlying ATTM-induced LUAD cell growth at low concentrations, we investigated mRNA m^6^A methylation, which has been involved in different cancer growth, like liver cancer (25), endometrial cancer (26), and leukemia (36). The m^6^A writer METTL3 has also been reported to promote lung cancer survival, growth, and invasion (29). We therefore speculated that the aberrant m^6^A methylation was involved in ATTM-induced growth of LUAD cells. By measuring m^6^A content in A549 cells, we found that the treatment with ATTM for 24 h significantly enhanced intracellular m^6^A content, while it did not significantly alter cell growth, suggesting m^6^A methylation occurs earlier than cell growth. Generally, m^6^A methylated mRNA can be synthesized via methyltransferase complex m^6^A writer, mainly consisting of METTL3, METTL14, and WTAP. Meanwhile, CH_3_- can also be erased from the RNA using FTO and ALKBH5. Therefore, the process is dynamic and reversible (37). The increased METTL3 and decreased FTO in the present TCGA analysis indicate that the dysregulated writer and/or eraser may be responsible for the increased m^6^A content in lung adenocarcinoma cells. This finding was supported by protein measurement in LUAD patients. Importantly, we found that the treatment with ATTM significantly augmented METTL3, but reduced FTO protein expression. ATTM could also upregulate METTL14 expression, which was not consistent with the TCGA analysis. We hypothesize that this difference may be due to the uncertainty of gene expression between mRNA level and protein level. Notably, for the increased METTL3 and decreased FTO, the present experiment matched the TCGA analysis. Such unique expressions, we believe, contribute to ATTM-induced m^6^A increase in A549 cells.

However, the roles of m^6^A in cancer biology are complicated and conflicting, i.e., tumor growth or anti-tumor (38). In addition, the binding of target mRNA with the writers or erasers is usually instantaneous. Therefore, it may be significant to examine the roles of m^6^A readers. To date, YT521-B homology (YTH) domain family of proteins, like YTHDF1, YTHDF2, YTHDF3, and YTHDC1, have been identified as m^6^A readers. YTHDF1-mediated mRNA spicing can increase translation efficiency, whereas YTHDF2-mediated mRNA decay will inhibit gene expression (21). The TCGA analysis indicates that the expression of YTHDF1, instead of YTHDF2/3 or YTHDC1, was markedly increased in LUAD tissues. In A549 cells, the elevated m^6^A mainly induced gene translation, evidenced by shMETTL3-mediated eIFs' downregulation (29). Western blot test showed that YTHDF1 expression could be upregulated by ATTM. Significantly, YTHDF1 siRNA inhibited basal and ATTM-induced cell growth, confirming the involvement of YTHDF1 in basal and environment-stimulated lung tumorigenesis.

As an m^6^A reader, it is necessary to discover YTHDF1's target genes. The TCGA analysis shows that PRPF6 (a small nuclear ribonucleic protein) is positively expressed with YTHDF1. The GEO analysis shows that PRPF6 can be attenuated through METTL3 knockdown (shGFP 1023 vs. shMETTL3 848). Additional analysis of MeT V2.0 m^6^A database indicates that m^6^A PRPF6 mRNA can be discerned and read by YTHDF1 in Hela cells via GRAC motif (R is G or A) (21). Actually, PRPF6 has been reported to promote lung cancer growth (39, 40). With these bioinformatics analyses and reports, PRPF6 is probably a target gene of YTHDF1. Importantly, the present experiment showed that ATTM treatment not only induced YTHDF1 expression, but also enhanced PRPF6 mRNA abundance. Both basal and ATTM-induced PRPF6 upregulation was significantly reduced by YTHDF1 knockdown, which was supported by previous reports (39, 40). As documented, the increased PRPF6 can alter the constitutive and alternative splicing of ZAK kinase, thereby activating cancer-related pathways, like AP-1, ERK, and JNK (41). In sum, it is believed that ATTM treatment upregulated METTL3/14 and downregulated FTO, raising intracellular m^6^A mRNA like PRPF6. Furthermore, these specific mRNAs were read by YTHDF1, and the spicing and translation of cancer growth-related genes were induced, thereby promoting LUAD tumor growth.

Significantly, we dissected the cause of ATTM-induced m^6^A methylation and cell growth. Copper chelating can exert anticancer effects through inhibiting angiogenesis (4, 6, 8). However, apart from copper chelating, ATTM can release H_2_S (10, 11). Studies have shown that the homeostasis of H_2_S is essential in organisms (42–46). In this study, we found that intracellular H_2_S level was markedly raised after ATTM treatment. Nevertheless, another copper chelator without H_2_S releasing ability, TETA, did not alter tumor cell growth. Direct H_2_S donation exerted similar effects to ATTM. The effects of ATTM or H_2_S could be abolished by CuSO_4_ or Zn(OAc)_2_. Besides H_2_S releasing, we found ATTM induced the expressions of H_2_S-production enzymes, which have been reported to participate in survival and chemoresistance of LUAD cells (18). Therefore, H_2_S is significant and necessary for ATTM-induced m^6^A methylation and lung cancer growth.

In healthy individuals, plasma free copper ion content is <20 μM (47), but for cancer patients, the recommended daily ATTM dosage was 90 mg (48). Therefore, the initial plasma ATTM should be much higher than that of free copper ions, so the free ATTM will release H_2_S during the clinical application (11). Of course, zinc application maybe overcomes ATTM's side-effects in LUAD therapy, while copper should not be recommended because of its tumorigenesis risk.

Interestingly, during the examination of VitB_12_ roles, it was found that VitB_12_ enhanced ATTM-induced LUAD cell growth (Supplementary Figure 1), unlike zinc or copper's inhibitory effects. However, both our study and previous reports suggest VitB_12_ is a H_2_S scavenger (34, 49). In fact, it has been documented that high VitB_12_ can raise the risk of lung cancer (17). H_2_S can also facilitate VitB_12_-induced SAM generation (16) that is the first substrate of METTTL3/14 complex. These findings further support H_2_S-induced m^6^A methylation promotes LUAD tumor growth.

**Figure 9:**
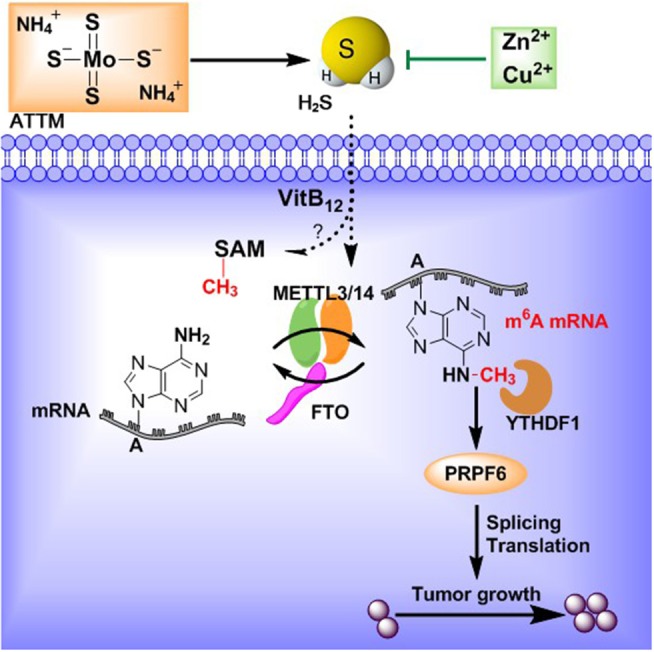
ATTM-produced H_2_S induces mRNA m^6^A methylation and promotes lung adenocarcinoma growth.

In conclusion, the present study demonstrated that ATTM can induce m^6^A methylation through upregulation of METTL3 and downregulation of FTO in LUAD cells. YTHDF1-mediated PRPF6 expression is probably a pivotal reason. The effects of ATTM are closely associated with H_2_S generation (Figure 9). For the first time, this work reveals a potential risk and mechanism for ATTM application in LUAD treatment. Meanwhile, this is the first report on the roles of H_2_S in m^6^A methylation.

## Data Availability Statement

The datasets generated for this study can be found in TCGA LUAD (http://ualcan.path.uab.edu/analysis.html), GSE76367 (https://www.ncbi.nlm.nih.gov/geo/).

## Ethics Statement

The studies involving human participants were reviewed and approved by Medical Ethics Committee of Guangzhou Medical University. The patients/participants provided their written informed consent to participate in this study.

## Author Contributions

CY, MX, JL, and JW designed the experiments and wrote the manuscript. XL, NL, SX, and XZ performed all the experiments and statistical analyses. LH and HZ analyzed the clinical data and provided the patient tissues. LD, MZ, and AH provided the critical suggestions. All the authors reviewed the final manuscript.

### Conflict of Interest

The authors declare that the research was conducted in the absence of any commercial or financial relationships that could be construed as a potential conflict of interest.

## Supplementary Material

Supplementary Figure 1 Effects of VitB_12_ on ATTM-induced A549 cell growth.
