# Supplementary figures and images for: Is Hydrogen Sulfide a Concern During Treatment of Lung Adenocarcinoma With Ammonium Tetrathiomolybdate?
Source: Front Oncol. 2020 Feb 28;10:234. doi: 10.3389/fonc.2020.00234 (PMC7061217; doi:10.3389/fonc.2020.00234)

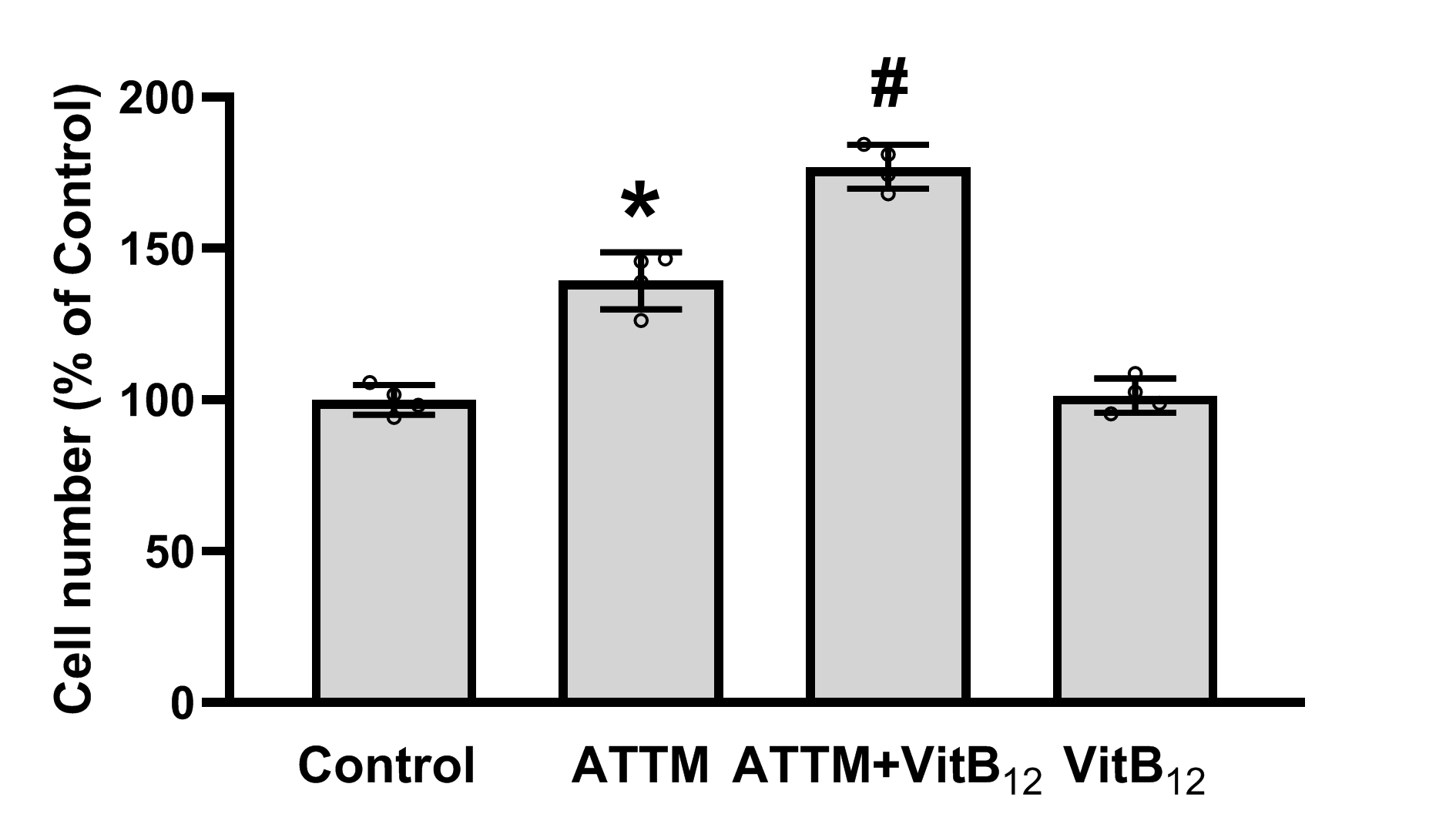

Supplement: Supplementary Figure 1 — Effects of VitB12 on ATTM-induced A549 cell growth. [file Image_1.TIF]
